# Predicting early infant diagnosis (EID) results for HIV exposed infants in a resource-limited setting using machine learning models: evidence from Amhara Public Health Institute data (2024/2025)

**DOI:** 10.1186/s12879-025-12508-8

**Published:** 2026-01-06

**Authors:** Zelalem Yitayal Melese, Mitiku Kassaw Takilo, Abraham Keffale Mengistu, Aynadis Worku Shimie, Gizaw Hailiye Teferi, Ashagrie Anteneh, Wubete Lule Ayalew, Sefefe Birhanu Tizie, Muluken Belachew Mengistie

**Affiliations:** 1 Department of Health Informatics, College of Medicine and Health Science, Debre Markos University, Debre Markos, Ethiopia; 2 Department of Midwifery, College of Medicine and Health Science, Debre Markos University, Debre Markos, Ethiopia

**Keywords:** Early infant diagnosis, Prevention of mother-to-child transmission, Machine learning, Prediction, HIV

## Abstract

**Introduction:**

Early infant diagnosis of human immunodeficiency virus is essential for timely intervention and treatment of exposed infants. Traditional diagnostic approaches often face logistical and cost-related challenges, leading to delayed results and reduced healthcare efficiency. Machine learning provides a promising alternative, enabling earlier identification of at-risk infants and more efficient allocation of healthcare resources.

**Methods:**

A cross-sectional study was conducted using early infant diagnosis data from the Amhara Public Health Institute, comprising 12,129 records with 12 features. Machine learning algorithms, including Decision Tree, Random Forest, Gradient Boosting Model, Logistic Regression, and Support Vector Machine, were trained and evaluated using Accuracy, Precision, Recall, F1-score, and Area Under the Curve (AUC). The Synthetic Minority Over-Sampling Technique was applied to address class imbalance.

**Results:**

The Gradient Boosting Model achieved the highest predictive performance with an AUC of 99.99%, followed by the Support Vector Machine with an AUC of 96.58%. Random Forest demonstrated the lowest performance with an AUC of 90.86%, highlighting its limitations in handling imbalanced datasets.

**Conclusion:**

Ensemble-based models, particularly the Gradient Boosting Model and the Support Vector Machine, significantly enhance the accuracy of HIV early infant diagnosis predictions among exposed infants. These models are therefore recommended as reliable tools for reducing both missed diagnoses and false-positive results in clinical practice.

## Introduction

Early Infant Diagnosis (EID) of HIV is a vital intervention to identify HIV infection in infants born to mothers living with HIV as early as “4–6 weeks up to 18 months” of age. Early detection enables the prompt initiation of antiretroviral therapy (ART), thereby improving survival, reducing disease progression, and supporting better health and development outcomes of affected children [1]. Due to maternal HIV antibodies persisting in infants for several months, standard antibody tests are not effective for diagnosing HIV in newborns; virological assays, which directly detect the virus, are essential for accurate and timely diagnosis [1].

HIV is a retrovirus that destroys the immune system by targeting CD4 + T cells. Without treatment, this leads to AIDS, leaving individuals vulnerable to opportunistic infections and cancer [1]. HIV spreads through blood (e.g., shared needles), semen, vaginal fluids (typically through unprotected sex), and breast milk [2, 3]. Vertical transmission—during pregnancy, childbirth, or breastfeeding—remains a significant concern for EID programs [3, 4].

Globally, MTCT remains a major contributor to new HIV infections, especially in resource-limited settings [5]. While ART has reduced MTCT rates, gaps in early diagnosis persist. EID, commonly performed via PCR testing, is essential for early ART initiation [6], but challenges like delays in results, logistical hurdles, and loss to follow-up hinder timely intervention [7].

In Ethiopia’s Amhara region, only 24% of HIV-positive pregnant women receive ART to prevent MTCT, and 10% of exposed infants diagnosed by EID are confirmed HIV-positive [8]. Current reliance on centralized PCR testing requires skilled personnel and specialized equipment, leading to delays that can miss the critical window for ART initiation [9, 10]. This inefficiency stresses the need for more accessible approaches to predict EID outcomes. Although statistical models have explored maternal ART, infant prophylaxis, and feeding practices [11].

Despite progress, HIV remains a major health crisis: in 2022, 39 million people lived with HIV, and about 630,000 died from AIDS-related causes [12]. Sub-Saharan Africa accounted for 54% of people living with HIV and 78% of AIDS-related deaths, driven by poverty, limited healthcare, social inequality, and conflict [13]. Other regions like Eastern Europe, Central Asia, and parts of Asia and Latin America also face growing epidemics in key populations [13].

Different cross-sectional studies conducted in Tanzania and Mozambique indicate that marital status, education level, area of residence, number of children since HIV diagnosis, and knowledge on EID are significantly associated with EID results [14, 15]. Moreover, A cross-sectional study conducted in 2023 in Buvuma District, Uganda, showed that none of the HIV-exposed infants (HEIs) received all the recommended tests within the prescribed time frames. Only 39.5% (120/304) received the first PCR test within the recommended 6–8 weeks, and just 6.1% (8/132) received the second PCR test at the recommended 58 weeks. However, 81.0% received the rapid diagnostic test within the recommended 126–168 weeks [16].

Based on different studies conducted previously in Ethiopia, Infants of mothers not attending ANC follow-up, Infant feeding practices, WHO clinical stage III, WHO stage IV, maternal ART use during pregnancy, and the place of birth are significantly associated with Early infant diagnosis of infants [17, 18]. A health institution-based retrospective cohort study conducted at St. Luke Hospital, Woliso town, Ethiopia, indicates that about 385 (90.4%) of the infants were HIV-negative at the final infection status. Infants of mothers not attending ANC follow-up (AOR = 5.28, 95% CI: 2.24–12.45), Infant feeding practices (AOR = 6.63, 95% CI: 1.23–35.56), WHO clinical stage III (AOR = 6.12, 95% CI: 1.87–20.07), and WHO stage IV (AOR = 10.23, 95% CI: 1.34–78.0) showed a significant association with infants acquiring HIV infection [17]. In addition, A multicenter retrospective cohort study conducted in public hospitals and health centers in Northwest Ethiopia found that a total of 35 infants were diagnosed with HIV infection, accounting for 13.2%. Predictors of early infant diagnosis were maternal prenatal care attendance, maternal ART use during pregnancy, and the place of birth [18].

Traditional methods may not fully capture complex data relationships. Machine learning (ML) offers potential to improve predictive accuracy by identifying non-linear patterns and incorporating diverse variables. This research seeks to develop and evaluate ML models for predicting EID outcomes, addressing gaps in timely diagnosis, particularly in settings where PCR testing is delayed or inaccessible [19]. The study is motivated by the absence of prior machine learning work on EID, the low availability of skilled personnel, and the existence of usable data. It aims to provide tools for better risk identification, ART initiation, and ultimately, improved survival and development for HIV-exposed infants. The findings could support data-driven policymaking, better resource allocation, and inspire further research on ML applications in global public health [11, 20].

This study seeks to determine the most accurate and reliable machine learning model for predicting early infant diagnosis (EID) outcomes among HIV-exposed infants in resource-limited settings. The findings are valuable for health policymakers and professionals, enabling data-driven decision-making to optimize EID programs, allocate resources more effectively, refine PMTCT strategies, and enhance monitoring and evaluation of interventions. Achieving these benefits requires investments in data infrastructure, integration of machine learning into clinical workflows, establishment of ethical guidelines, and policy adjustments to prioritize interventions identified as most impactful by the predictive model. Additionally, the results have important implications for clients, including pregnant women, infants, and families, by improving timely access to EID services and treatment initiation. The model’s ability to identify high-risk infants can guide targeted interventions, while its data-driven approach helps reduce HIV/AIDS-related stigma and empowers families with knowledge about risk factors, testing, and treatment. Ultimately, this promotes greater awareness of EID benefits and enhances access to essential support services.

## Methods

### Study design and setting

A cross-sectional study design was employed to predict early infant diagnosis (EID) outcomes among HIV-exposed infants using machine learning. The study utilized EID data from the Amhara Public Health Institute (APHI) collected between 2016 and 2024. APHI is a central public health institution in the Amhara region of Ethiopia, playing a key role in health surveillance, data management, laboratory services, and program implementation. The institute is located in Bahir Dar, approximately 550 km from Addis Ababa and 255 km from Debre Markos.

### Source and study population

The source population and study population were identical, consisting of infants aged 0 to 104 weeks. The study included all HIV-exposed infants who were tested and had a recorded final EID result.

### Data source

The data for this study were obtained from the Amhara Public Health Institute (APHI) EID/Viral Load database, covering the period from 2016 to 2024. The dataset was provided in Excel format, containing 12,129 records and 19 features. Of these 19 features, only 11 were present in the original APHI dataset. The rest 8 features underwent feature engineering using adding and subtracting techniques of the existing feature data. These engineered features were “delay start on ART”, “age at tested”, “delays sample transportation”, “delay sample collection”, “delay sample transferring”, “delay to sample to drawn”, “delay on start NVP”, and “age in weeks range”.

### Variables/features

#### Outcome variable

EID result (Positive-1/ Negative-0).

#### Predictor variables

Demographic data (age, age at tested, sex, age in weeks range, and age at tested in weeks range), HIV care and support of HIV exposed infants and their mothers related variables (delay start on ART, delay sample transportation, delay sample collection, delay to test, delay to nevirapine started, delay on sample transfer, delay to sample to drawn, PMTCT intervention, type of sample, daily nevirapine start from birth, daily nevirapine for 6 weeks, mothers ART initiated, mothers gain preventive ART, mothers ART unknown).

### Model development

**Fig. 1:**
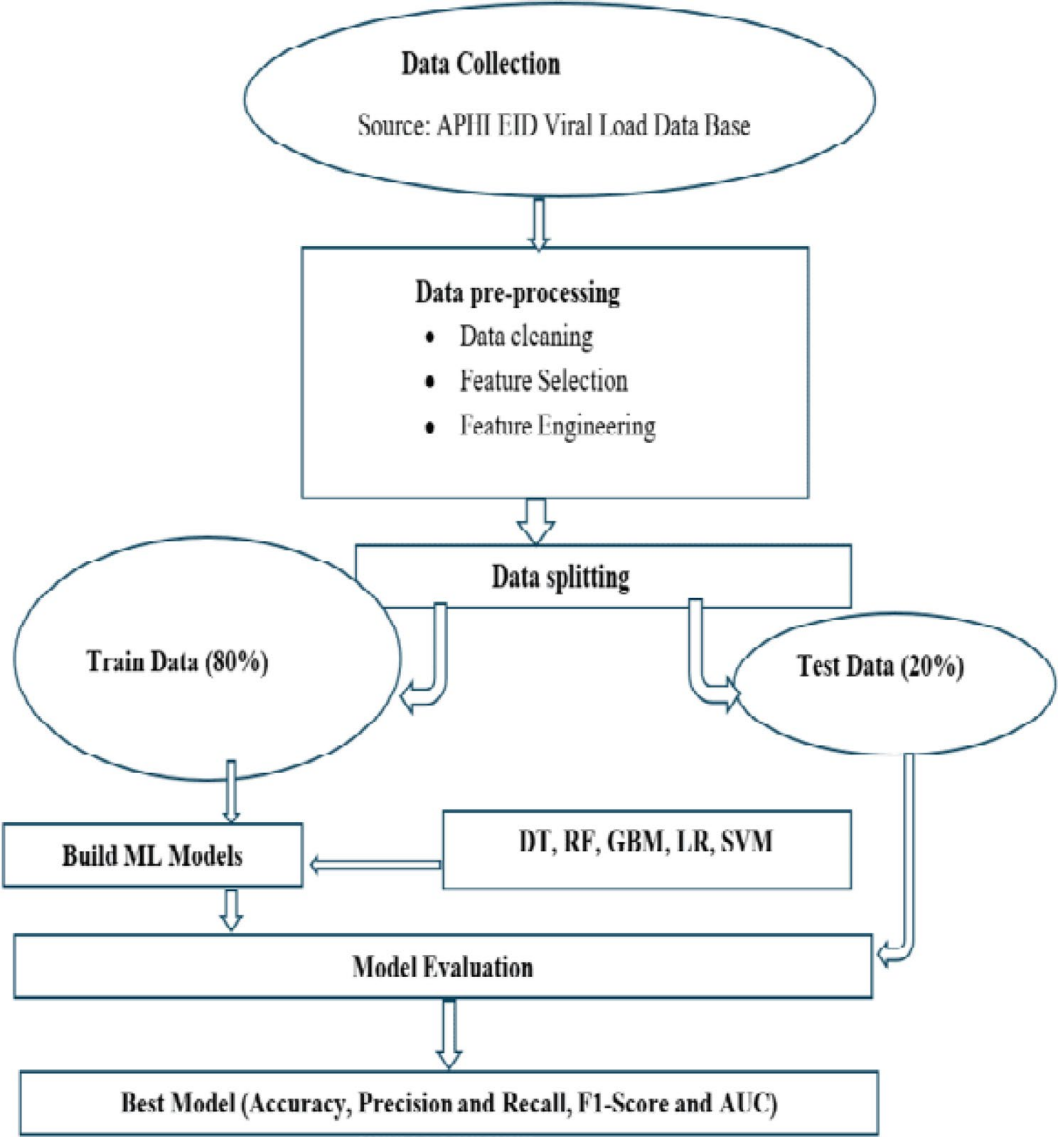
Model framework to predict EID results for HIV exposed infants

Once the data was prepared and split into training and testing sets, appropriate models were selected for training. The predictive algorithms used in this study included Decision Tree, Random Forest, Gradient Boosting Model, Logistic Regression, and Support Vector Machine, chosen based on evidence from previous research (Fig. 1).

Logistic Regression is a predictive technique used when the outcome variable is discrete, typically binary, and it models the relationship between the dependent variable and one or more independent variables. A Decision Tree is a tree-like structure that represents decisions and their possible outcomes, useful for supporting decision-making. Gradient Boosting and Random Forest are ensemble-based methods that perform effectively with datasets containing both categorical and continuous features. Support Vector Machine is well-suited for high-dimensional data, classifying instances by finding an optimal decision boundary, which may be a line or a multidimensional hyperplane. The Gradient Boosting Model is another ensemble learning method that builds predictive models by sequentially combining multiple decision trees to improve performance. Additionally, SHapley Additive exPlanations (SHAP) was conducted to illustrate how each feature contributes to the infant diagnosis predictions and enhance the interpretability of the models.

### Class balancing

The total dataset consisted of 12,129 samples (both negative and positive cases), encompassing both the training and test sets. The dataset was first split into training (80%) and test (20%) sets while preserving the original class distribution. In the training set, the majority class (negative EID) included 9,685 samples (80% of 12,107), and the minority class (positive EID) included 17 samples (80% of 22).

**Fig. 2:**
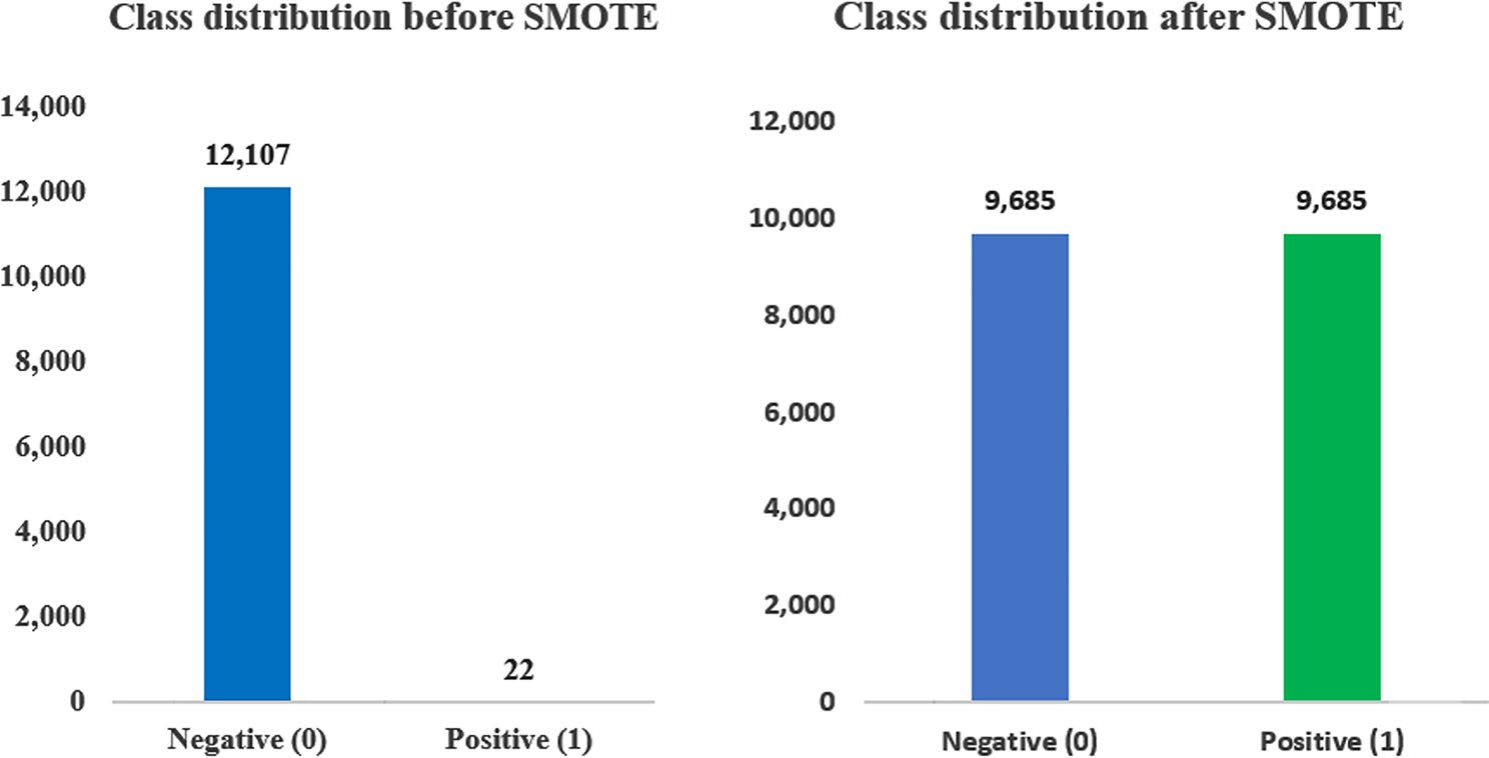
Class distribution before and after SMOTE

To address the severe class imbalance, SMOTE was applied only to the minority class in the training set. SMOTE generated synthetic positive EID samples until the number of minority class samples matched the majority class. After resampling, the training set became balanced, with 9,685 samples in each class, while the test set remained unchanged (Fig. 2).

### Data preprocessing

Data preprocessing, including data cleaning, exploratory analysis, normalization, dimensionality reduction, and feature engineering, was performed to enhance model performance using Python libraries, Power BI, and Excel. In this study, the primary data preparation steps included data cleaning, dimensionality reduction, and data splitting. These procedures involved techniques such as feature selection, dataset partitioning, and hyperparameter tuning to optimize model performance.

#### Feature selection

Feature selection is a crucial step prior to model development. In this study, three strategies were employed: Recursive Feature Elimination (RFE), feature importance scoring, and correlation analysis, aimed at improving both model performance and interpretability for the EID dataset.

Initially, feature importance analysis identified the most influential predictors, such as breastfeeding status, test delay, and maternal ART history, prioritizing variables that enhance classification accuracy. Next, correlation analysis addressed multicollinearity, which can cause instability when predictors convey overlapping information. Managing these correlations ensures reliable relationships among features, which is critical for accurately detecting HIV-positive infants.

RFE was combined with SMOTE to further improve the detection of the minority class. Given the inherent imbalance in EID datasets, fewer HIV-positive cases were compared to negative SMOTE-generated synthetic samples to balance the dataset, while RFE systematically removed weak or redundant features, retaining only the most impactful predictors. This approach reduces overfitting to the majority class and strengthens the model’s ability to identify HIV-positive infants.

**Fig. 3:**
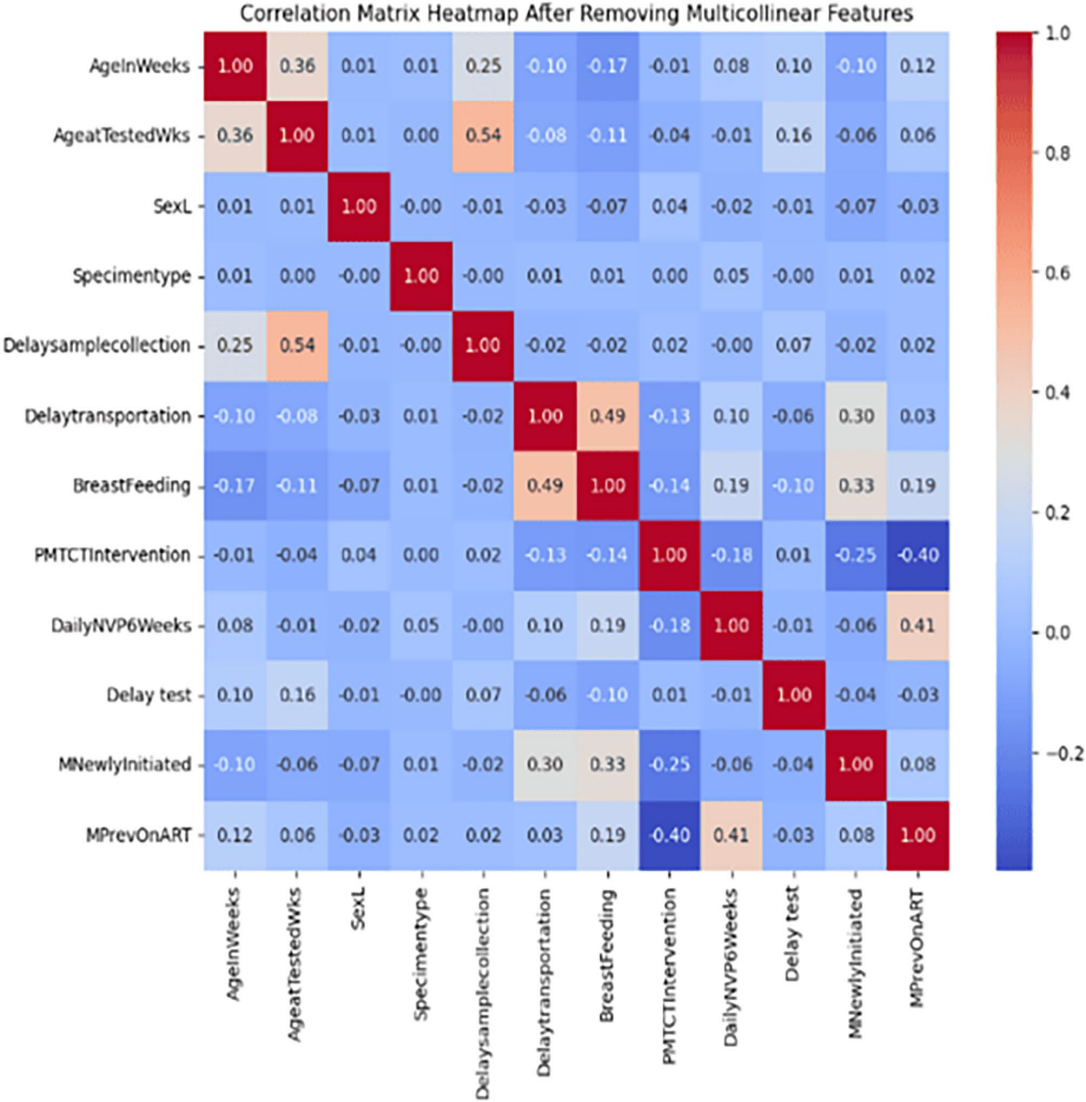
Heat map plot showing the correlations among selected features of EID

Correlation matrix analysis showed strong positive correlations among some age-related features (AgeInWeeks and LAgeInWeeks: 0.95; AgeatTestedWks and LAgeatTestedWks: 0.92). Some negative correlations were observed, such as between breastfeeding and certain delay variables. The heatmap visualization facilitated the identification of multicollinearity, which informs feature selection and modeling. To manage this collinearity, ‘LAgeInWeeks’, ‘LAgeatTestedWks’, ‘Delaytosampletransfer’,‘Lengthofdelaysampletodrow’, ‘MARTUnknown’, ‘DelayNVPstarted’, and ‘DailyNVPBirth’ features were dropped from the analysis (Fig. 3).

#### Data splitting

Every machine learning algorithm requires training and testing (or validation) data to learn patterns and evaluate performance on unseen data. In this study, a simple 80/20 split was applied, with 80% of the data used for training and the remaining 20% reserved for testing the model.

#### Parameter tuning or hyper-parameter optimization

Hyperparameter tuning refers to the process of identifying the optimal set of hyperparameter values for a specific machine learning algorithm on a given dataset. This process aims to determine the values that maximize the model’s performance on a validation set or through cross-validation.

### Model training

In this study, following data cleaning, the dataset was split into 80% for training and 20% for testing. Feature selection was performed using the Gradient Boosting Model to identify the most relevant predictors for EID. Five classifiers, namely Logistic Regression (LR), Decision Tree (DT), Random Forest (RF), Gradient Boosting Model (GBM), and Support Vector Machine (SVM), were then trained on the training set. These algorithms were chosen based on previous studies that applied machine learning techniques for classification tasks. The performance of each model was subsequently evaluated on the test dataset. Furthermore, an early stopping strategy in GBM was applied to prevent overfitting and to ensure that the model maintains good generalizability to unseen data, improves predictive reliability, and reduces the risk of fitting noise in the training dataset.

### Model evaluation metrics

The models were evaluated using multiple performance metrics, including Accuracy, Precision, Recall, F1-Score, and AUC.

## Results

### Description of demographic characteristics of HIV exposed infants

The infants’ age at enrollment was 12.61 ± 17.71 weeks (mean ± standard deviation). More than half of the infants were female (52%), with males accounting for the remaining 48%. At enrollment, most infants were aged ≤ 6 weeks (57.5%), followed by those aged 7–12 weeks (19.9%) and 13–24 weeks (18.8%). Correspondingly, the majority of infants underwent HIV testing at 7–12 weeks of age (59.4%), while 15.8% were tested at ≤ 6 weeks and 21.3% between 13 and 24 weeks. Regarding PMTCT services, nearly two-thirds of the infants (62%) received optimal PMTCT interventions, whereas 38% received suboptimal care (Table 1).

**Table 1:** Description of demographic characteristics of HIV exposed infants

Variables	Category	Percent (%)
Sex	Female	52%
	Male	48%
Age (Weeks)	≤ 6 Weeks	57.5%
	7–12 Weeks	19.9%
	13–24 Weeks	18.8%
	> 24 Weeks	3.8%
Age at tested (Weeks)	≤ 6 Weeks	15.8%
	7–12 Weeks	59.4%
	13–24 Weeks	21.3%
	> 24 Weeks	3.5%
PMTCT Intervention	Optimal	62%
	Suboptimal	38%

### Machine learning models’ evaluation results

#### Comparison of model accuracy

**Fig. 4:**
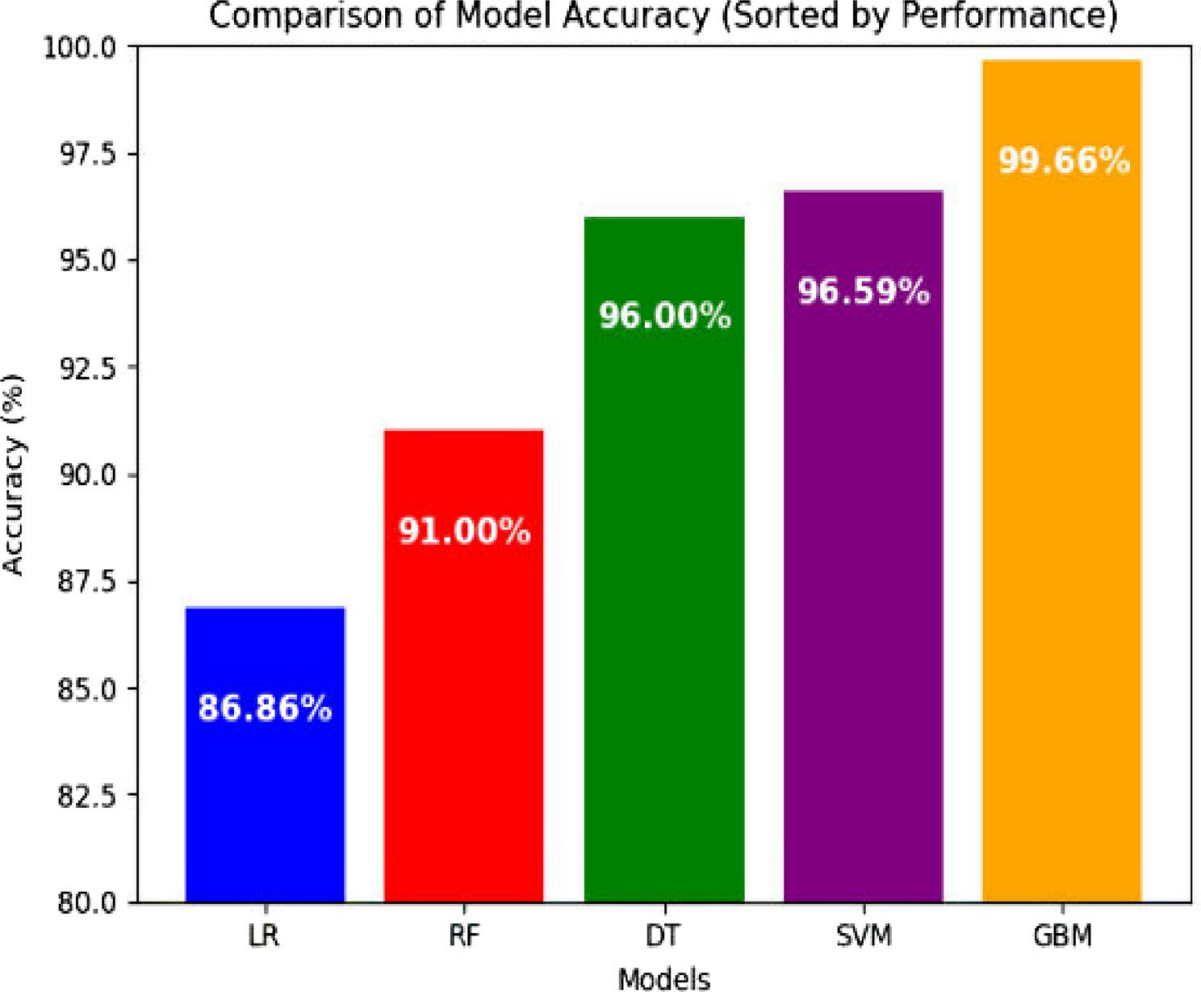
All model performance in accuracy to predict EID results for HIV exposed infants

Model evaluation results indicate that the Gradient Boosting Model (GBM) outperformed all other classifiers, achieving an impressive 99.66% accuracy in predicting EID, followed by the Support Vector Machine with 96.59% accuracy (Fig. 4).

#### Comparison of model precision

**Fig. 5:**
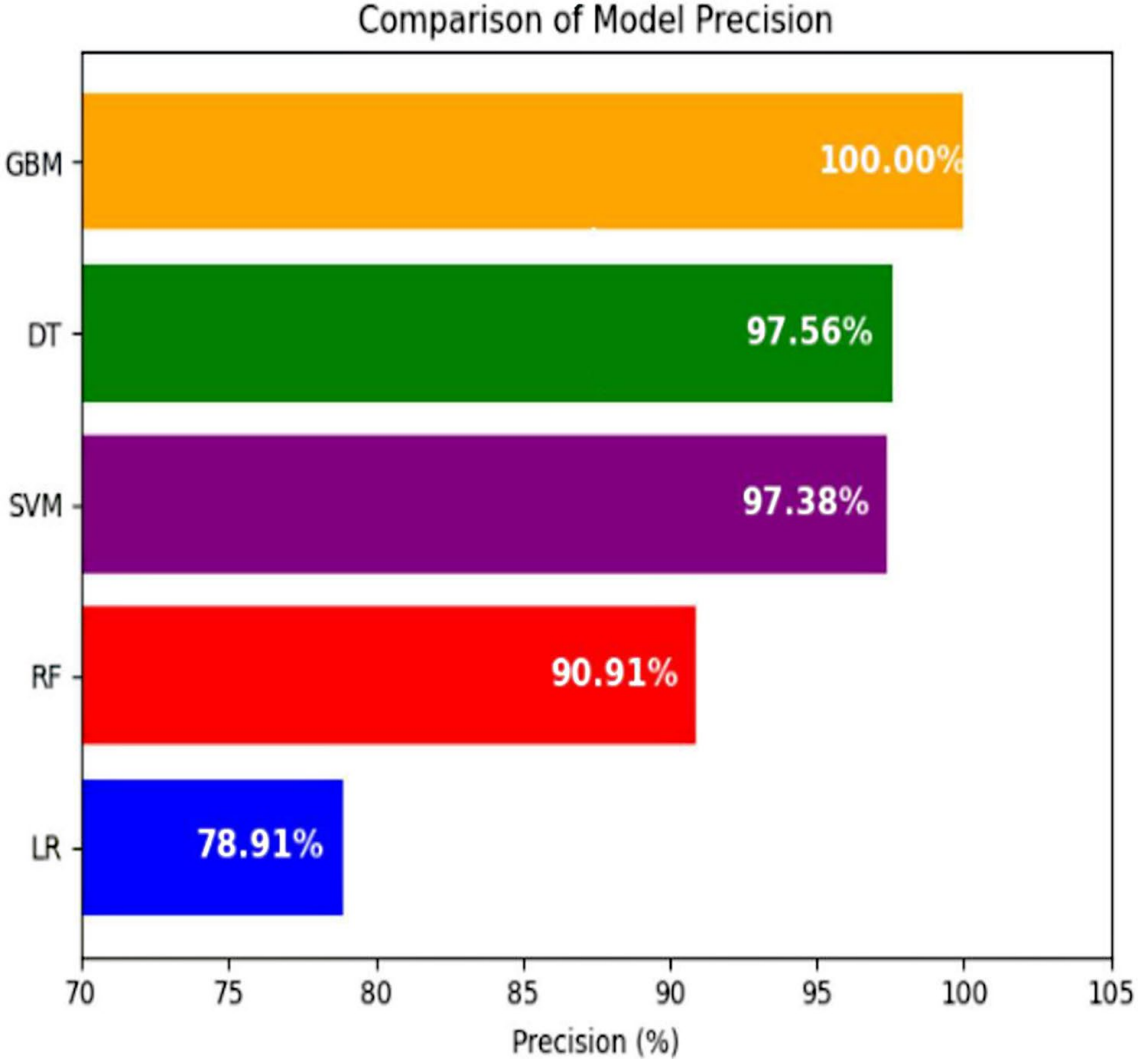
All model performance in precision to predict EID results for HIV exposed infants

Among all models, GBM (100%), DT (98.25%), and SVM (97.51%) achieved the highest precision, reflecting strong performance in correctly identifying negative cases. In contrast, Logistic Regression and Random Forest showed lower precision scores (Fig. 5).

#### Comparison of model F1-score

Among all models, Random Forest, SVM, and GBM achieved perfect or near-perfect F1-scores, while Logistic Regression and Decision Tree struggled to balance precision and recall, resulting in an F1-score of 0 (Table 2).

**Table 2:** All model performance in F1-score to predict EID results for HIV exposed infants

Model	F1- Score (%)
GBM	99.66
SVM	96.50
DT	95.24
RF	91.74
LR	88.21

#### Comparison of model recall (sensitivity) score

**Fig. 6:**
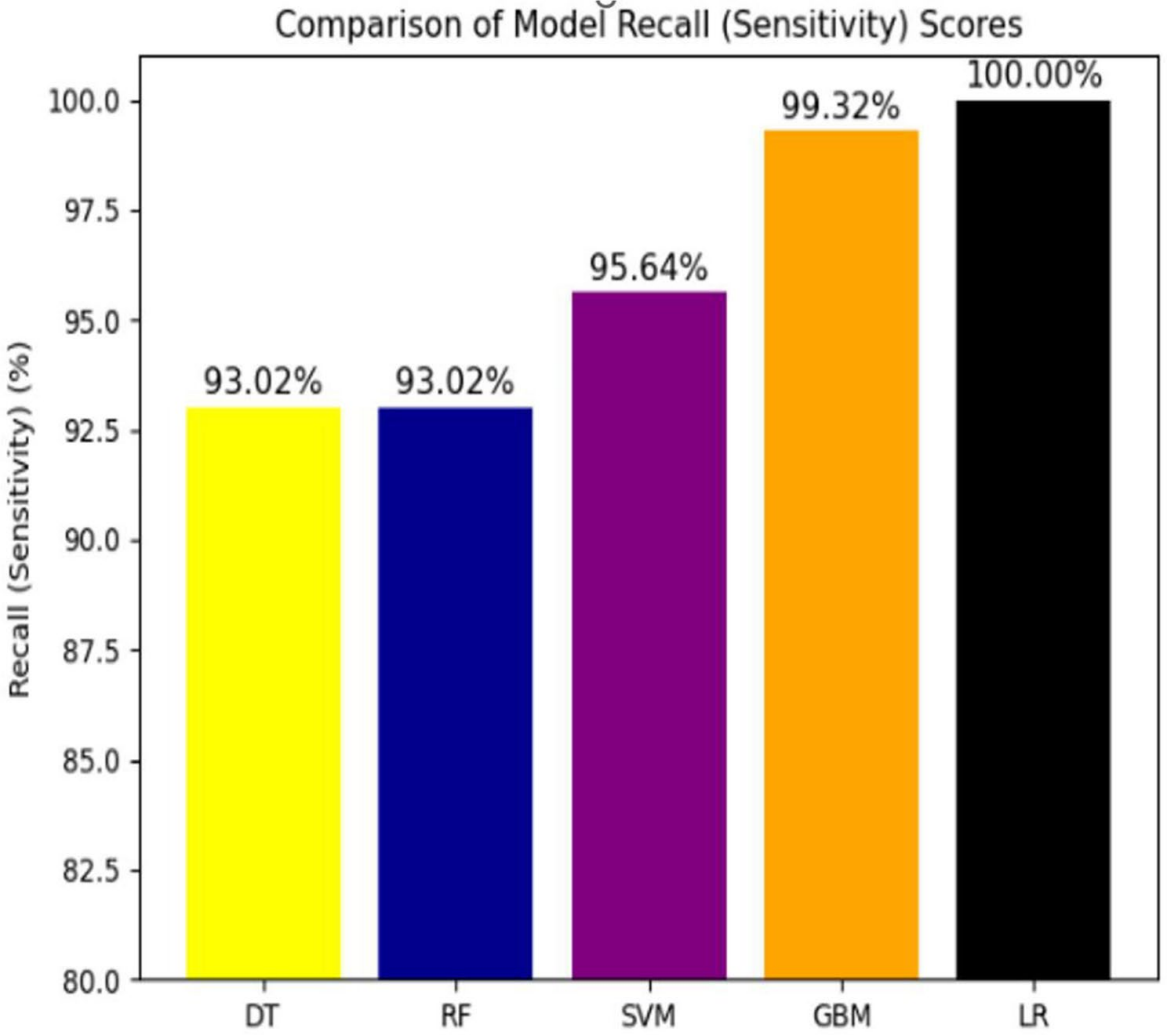
All model performance on recall to predict EID results for HIV exposed infants

Among all machine learning models, Logistic Regression (100%) and the Gradient Boosting Model (99.32%) demonstrated the highest recall (sensitivity) scores compared to the other models (Fig. 6).

#### Comparison of model AUC score

Among all the ML models, the Gradient Boosting Model (99.99%) and the Support Vector Machine (96.58%) achieved the highest AUC scores for predicting early infant HIV diagnosis. In contrast, Logistic Regression (93.32%) and Random Forest (90.86%) showed comparatively lower AUC values (Table 3).

**Table 3:** All model performance AUC scores to predict EID results for HIV exposed infants

S. N	Model	AUC Score (%)
1	GBM (Gradient Boosting Machine)	99.99
2	SVM (Support Vector Machine)	96.58
3	DT (Decision Tree)	95.61
4	LR (Logistic Regression)	93.32
5	RF (Random Forest)	90.86

### Confusion matrix

**Fig. 7:**
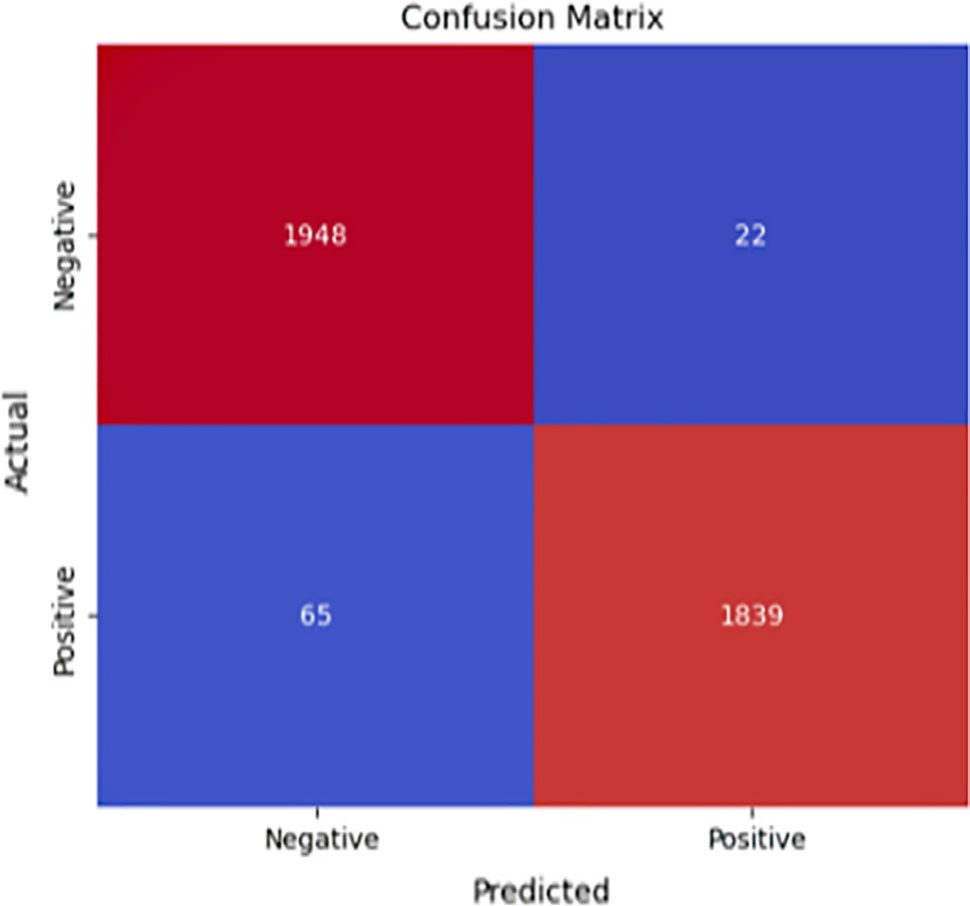
Confusion matrix for GBM model performance metrics to predict EID results

The confusion matrix demonstrates the GBM model’s high sensitivity and low overall error rate, The GBM model was trained on the resampled balanced dataset produced using SMOTE. After training, the model was tested on a 20% test split from the balanced dataset (3,874 samples). The model correctly classified 1,948 HIV-negative infants (True Negatives) and 1,839 HIV-positive infants (True Positives). While misclassifications were minimal, 22 HIV-negative infants were incorrectly predicted as positive (False Positives), and 65 HIV-positive infants were misclassified as negative (False Negatives) (Fig. 7).

### Feature importance

Feature importance analysis identified the predictors that most strongly influenced the GBM model’s decisions. Variables with the highest feature importance score, such as delayed testing, delayed sample collection, infant age in weeks, specimen type, and breastfeeding, had the greatest impact on predicting EID outcomes (Table 4).

**Table 4:** Feature importance for GBM model performance metrics to predict EID results

S. N	Feature	Importance Score
1	Delaytest	0.3539
2	Delaysamplecollection	0.1865
3	AgeInWeeks	0.1225
4	Specimentype	0.0846
5	BreastFeeding	0.0827
6	DailyNVP6Weeks	0.0460
7	Delaysampletransportation	0.0425
8	MPrevOnART	0.0389
9	PMTCTIntervention	0.0237
10	MNewlyInitiated	0.0231
11	SexL	0.0191
12	AgeatTestedWks	0.0187

### Explainable AI (XAI) integration

**Fig. 8:**
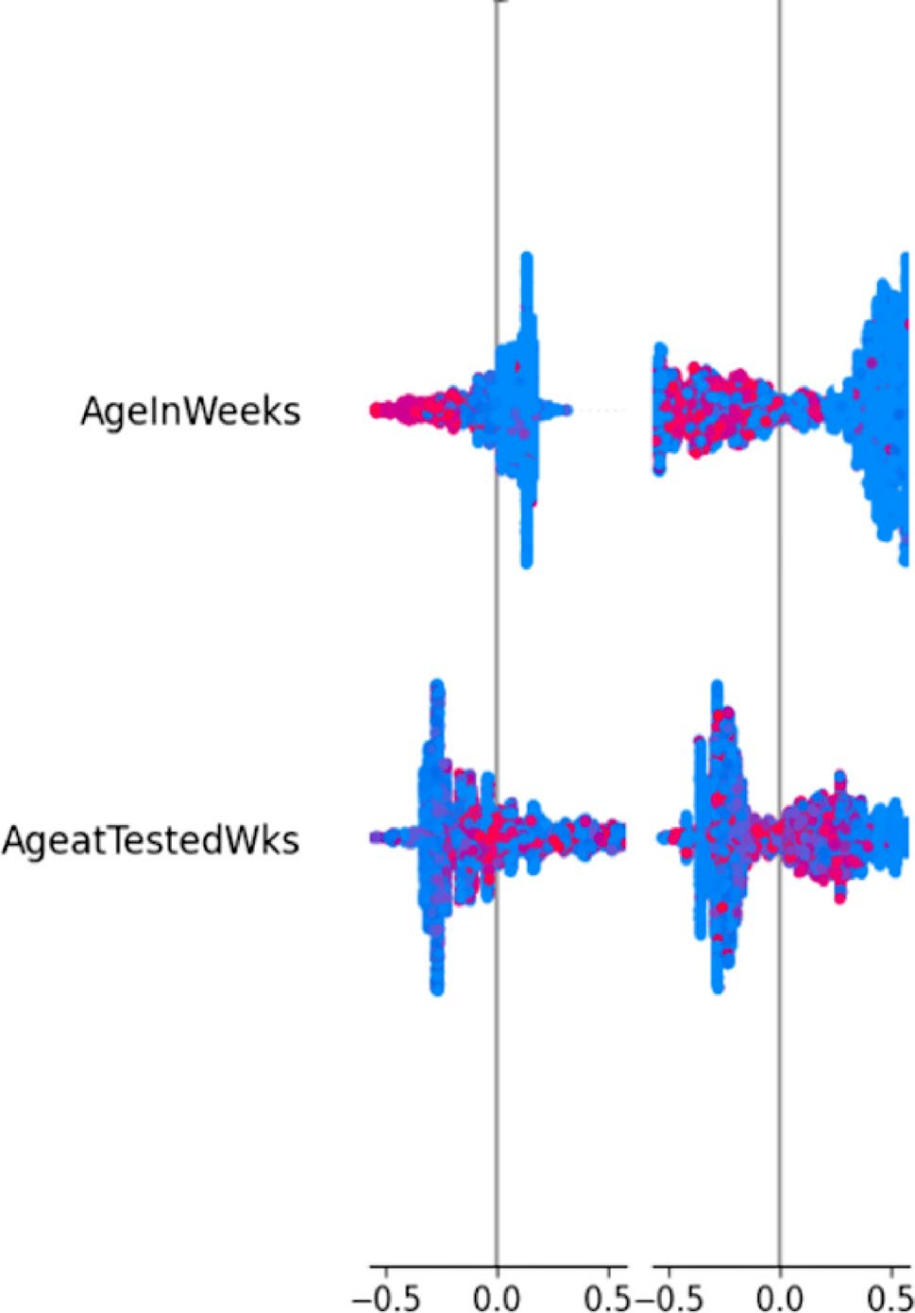
SHAP analysis plot

This study employs the SHAP explainable AI technique to illustrate the contribution of each feature to infant diagnosis predictions, thereby improving model interpretability. The SHAP interaction plot reveals that both “AgeInWeeks” and “AgeatTestedWks” influence the final EID outcome in a nonlinear manner. Higher values of either variable are mostly represented by blue points with positive SHAP interaction values, suggesting that older infants or later testing dates are associated with a higher probability of a positive EID result. Conversely, lower values appear as pink/red points near zero or negative interaction values, indicating that younger infants or earlier testing contribute less to the predicted risk (Fig. 8).

## Discussion

Notably, the GBM demonstrated strong predictive capability, achieving an accuracy of 99.66%, a recall of 99.32%, a precision of 100%, and an F1-score of 99.66%. Its near-perfect AUC of 99.99% further confirms the model’s exceptional ability to distinguish between HIV-positive and HIV-negative cases. These findings align with previous studies highlighting the effectiveness of gradient boosting methods in highly sensitive healthcare settings, especially where accurate minority-class detection is essential [9, 21]. Based on previous studies conducted before, the Gradient Boosting Model performed well, with the training accuracy (99.05%) slightly higher than the test accuracy (98.66%), indicating mild overfitting. To address this, early stopping was applied as a regularization strategy. After implementing early stopping, the model showed notable improvements: accuracy increased to 99.66%, recall to 99.32%, precision reached 100%, and the AUC rose to 99.99%, demonstrating stronger generalization. The gap between training and test accuracy decreased from 0.39% to 0.22%, confirming reduced overfitting. These improvements highlight early stopping as an essential technique for optimizing GBM performance in healthcare contexts. Overall, the final results affirm GBM’s exceptional classification power, particularly in settings with pronounced class imbalance, making it a highly suitable tool for early HIV detection among exposed infants [21, 22].

The Support Vector Machine classifier was trained using a balanced dataset created through the Synthetic Minority Over-sampling Technique (SMOTE) to correct the initial class imbalance. The data was then divided into 80% for training and 20% for testing. When evaluated on the test set, the SVM demonstrated strong predictive performance, achieving an accuracy of 96.59%, a precision of 97.51%, a recall of 95.64%, and an F1-score of 96.50%. It also recorded a specificity of 97.51% and an AUC of 96.58%, indicating a high level of discrimination between HIV-positive and HIV-negative infants [23]. These metrics show that the model effectively balanced the reduction of both false positives and false negatives. The recall value is particularly crucial for HIV diagnosis, as it measures the model’s ability to correctly detect nearly all true HIV-positive cases—an essential requirement in clinical settings where missed diagnoses can lead to delayed treatment and heightened health risks. This outcome is consistent with findings reported in previous studies [23].

The Decision Tree (DT) classifier showed moderate performance in predicting HIV status among exposed infants, achieving an overall accuracy of 96%, an F1-score of 95.24%, and an AUC of 95.61%. It correctly classified 40 HIV-positive and 56 HIV-negative cases but misidentified three positive infants—an important limitation given the clinical significance of early HIV detection. Although the model is valued for its simplicity, interpretability, and computational speed, it displayed clear signs of overfitting, with a training accuracy of 99.25% compared to a much lower test accuracy of 90%. Even after hyperparameter tuning, the model’s performance deteriorated further, with test accuracy dropping to 91% and specificity declining to 89.13% [21].

The Random Forest (RF) model demonstrated strong classification performance, benefiting from its ensemble architecture. It achieved an F1-score of 91.74%, an AUC of 90.86%, and a recall of 92.59%, reflecting solid capability in identifying minority-class (HIV-positive) cases. The RF model also showed stability and good interpretability, particularly after hyperparameter tuning, which optimized factors such as the number of trees and their maximum depth. These adjustments improved its reliability across both the training and test sets. Prior research has similarly emphasized the adaptability of Random Forest in clinical settings with imbalanced data, noting its ability to deliver a strong balance between predictive accuracy and computational efficiency [24].

Logistic Regression (LR) demonstrated the lowest predictive performance among the models based on evaluation metrics. Its relatively weaker results, reflected in lower F1-scores and AUC values, align with previous healthcare studies involving imbalanced datasets [25]. Unlike ensemble methods, LR struggled to correctly identify negative cases (74.16%), even though it achieved an AUC of 93.32%. This observation is consistent with prior research, which highlights that conventional logistic regression often underperforms in situations with severe class imbalance and may require threshold adjustments or integration with ensemble approaches to enhance sensitivity [26].

Multiple studies have identified class imbalance as a major challenge in HIV prediction models. To mitigate this imbalance during model development, SMOTE was applied to enhance minority class learning. Although SMOTE can improve model sensitivity, it generates synthetic observations derived from existing data and may not fully represent the clinical, virological, and programmatic heterogeneity encountered in routine WHO-recommended EID settings. Consequently, predictive performance achieved on SMOTE-balanced data may not directly translate to real-world EID implementation, where HIV-positive cases remain rare. These findings should therefore be interpreted with caution, and external validation using independent datasets from routine EID programs is essential before considering integration into WHO-aligned clinical workflows. Previous similar research has shown that models trained on imbalanced HIV datasets often exhibit low recall and precision for the minority class, a pattern that was also observed in the Decision Tree and Logistic Regression models in this study [22]. The application of SMOTE substantially improved model balance, consistent with prior findings where SMOTE enhanced classification performance in medical datasets [27].

Key predictors identified in this study included delayed testing, delayed sample collection, infant age, specimen type, and breastfeeding status. These findings are consistent with other studies, which highlighted maternal ART status and infant age at testing as important factors affecting EID outcomes [8, 28]. Notably, this study emphasizes the impact of delays in sample testing, echoing previous research that linked logistical delays to missed diagnoses and postponed treatment [29].

The integration of machine learning with explainable AI (XAI) in early infant diagnosis among HIV-exposed infants provides a robust approach to improving the detection of HIV infection. Explainable AI techniques, such as SHAP, are incorporated to enhance the transparency, interpretability, and trustworthiness of model predictions. SHAP analyses indicate that factors such as infant age in weeks and age at diagnosis are key interacting variables influencing EID outcomes. A 2025 study using interpretable ML models demonstrated that combining ML with XAI can substantially improve clinical decision-making, optimize diagnosis timing, and support personalized patient care [30]. Additionally, several XAI applications have been reported in clinical contexts, including the diagnosis of febrile illnesses [31] and the detection of tropical diseases in adult females [32].

A systematic review conducted in 2025 found that Shapley Additive Explanations (SHAP) and Local Interpretable Model-agnostic Explanations (LIME) are the most widely used XAI techniques in the medical field for disease prediction across various countries [33]. Additionally, explainable AI models have been applied to clarify fever diagnoses, such as malaria and typhoid, for healthcare professionals. These models enhance trust and transparency in medical decision-making by identifying the symptoms that most influenced the model’s predictions and providing clear, interpretable explanations [34]. Furthermore, an ensemble and explainable machine learning approach was implemented in preterm birth prediction [35], tracking fetal kicks and movements [36], and gestational diabetes mellitus prediction [37].

## Conclusion

The findings of this study show that the Gradient Boosting Machine outperformed the other models, achieving high levels of accuracy, precision, and recall almost with a perfect capacity to distinguish between HIV-positive and HIV-negative infants. The Support Vector Machine also demonstrated strong predictive ability, particularly in accurately identifying HIV-positive cases. However, the generalizability of these models is limited, as they were developed using data exclusively from the Amhara region and may not perform comparably in settings with different health system characteristics, sociodemographic conditions, HIV prevalence rates, testing protocols, or healthcare infrastructure. Future research should prioritize prospective validation and evaluation across diverse regions and healthcare settings to enhance generalizability and support the practical implementation of findings.

## Data Availability

All relevant data are available within the paper and corresponding author.

## Abbreviations

AIDS: Acquired Immune Deficiency Syndrome
APHI: Acquired Immune Deficiency Syndrome
AUC: Area Under the Curve
DT: Decision Tree
EID: Early Infant Diagnosis
GBM: Gradient Boosting Model
HIV: Human Immunodeficiency Virus
LR: Logistic Regression
ML: Machine Learning
PCR: Polymerase Chain Reaction
PMTCT: Prevention of Mother-to-Child Transmission
RF: Random Forest
SVM: Support Vector Machine
SHAP: Shapley Additive Explanations
SMOTE: Synthetic Minority Over-sampling Technique
